# Emerging Robust Polymer Materials for High-Performance Two-Terminal Resistive Switching Memory

**DOI:** 10.3390/polym15224374

**Published:** 2023-11-10

**Authors:** Bixin Li, Shiyang Zhang, Lan Xu, Qiong Su, Bin Du

**Affiliations:** 1 School of Physics and Chemistry, Hunan First Normal University, Changsha 410205, China; lbxin86@hotmail.com (B.L.);; 2 Shaanxi Institute of Flexible Electronics (SIFE), Northwestern Polytechnical University (NPU), Xi’an 710072, China; 3 School of Physics, Central South University, 932 South Lushan Road, Changsha 410083, China; 4 School of Materials Science and Engineering, Xi’an Polytechnic University, Xi’an 710048, China

**Keywords:** polymer, biomaterials, resistive switching memory, data storage, memristors

## Abstract

Facing the era of information explosion and the advent of artificial intelligence, there is a growing demand for information technologies with huge storage capacity and efficient computer processing. However, traditional silicon-based storage and computing technology will reach their limits and cannot meet the post-Moore information storage requirements of ultrasmall size, ultrahigh density, flexibility, biocompatibility, and recyclability. As a response to these concerns, polymer-based resistive memory materials have emerged as promising candidates for next-generation information storage and neuromorphic computing applications, with the advantages of easy molecular design, volatile and non-volatile storage, flexibility, and facile fabrication. Herein, we first summarize the memory device structures, memory effects, and memory mechanisms of polymers. Then, the recent advances in polymer resistive switching materials, including single-component polymers, polymer mixtures, 2D covalent polymers, and biomacromolecules for resistive memory devices, are highlighted. Finally, the challenges and future prospects of polymer memory materials and devices are discussed. Advances in polymer-based memristors will open new avenues in the design and integration of high-performance switching devices and facilitate their application in future information technology.

## 1. Introduction

In the current big data era, there is a growing demand for exploring data-storage technologies [1,2]. Therefore, various innovative data storage devices have been developed, such as dynamic random-access memory (DRAM), static random-access memory (SRAM), and flash memory. These memory devices are mainly based on conventional Si-based technologies, which suffer from physical miniaturizing limitations due to technical complexity and high cost [3,4,5]. They cannot fulfill the ever-increasing demands of high data density and fast switching speed. Therefore, striving for next-generation information storage solutions with faster speed, higher density, and lower power is imperative.

Recently, phase change memory (PCM), spin-transfer torque magnetoresistive random-access memory (STT-MRAM), and resistive random-access memory (RRAM) have been developed as next-generation non-volatile memory technologies [6,7,8,9,10,11,12,13]. PCM and STT-MRAM suffer from long write latency and low reliability, respectively. In contrast, RRAM devices with the advantages of low power consumption, high scalability, simple structure, easy fabrication, and low cost are considered promising candidates for future memory technology. The International Semiconductor Technology Roadmap identifies RRAM as one of the new memory technologies with the greatest potential for commercialization. Generally, RRAMs are fabricated as a sandwiched structure with a functional active layer sandwiched between two electrodes. The resistance states can be switched between high-resistance state (HRS, OFF state) and low-resistance state (LRS, ON state) in response to an external electrical stimulus, which is equivalent to “0” to “1” binary conversion. When more than two resistance states show in one material (e.g., “0”, “1”, “2”), multilevel storage can be expected, and it will increase the storage capacity within one memory cell exponentially [14,15,16].

To date, various functional materials have been explored for RRAMs, including organic materials, inorganic oxide, and organic–inorganic hybrid materials. The inorganic oxide-based RRAMs exhibit remarkable and stable memory characteristics, while they are limited by non-flexibility, non-recyclability, and environmental unfriendliness in the application of future wearable electronics. Organic polymer materials with the advantage of designable molecular structures, low costs, intrinsic flexibility, solution processability, 3D-stacking capability, and good biocompatibility, have been developed as favorable devices in next-generation memory technology [17]. Numerous efforts have been made to seek high-performance memory devices with a large ON–OFF ratio, low operation voltage, long retention time, as well as high endurance. In particular, the intrinsic flexibility and softness of polymers, especially biomacromolecules, make them ideal for stretchable and wearable electronics in the artificially intelligent lifestyle of the future [18,19,20]. These devices are mainly based on polymer composites and single-polymer materials. Compared with polymer composites, single-polymer materials can remove the phase separation problem. In order to explain the memory mechanisms, space charge limited current (SCLC), charge transfer, charge trapping/detrapping, filament conduction, and conformational change have been proposed [6,21,22].

In this review, we focus on the recent advances in polymer materials for resistive switching (RS) memory applications and aim to provide comprehensive concepts to develop highly efficient devices for next-generation information technology. First, this review gives a brief introduction on the structure of memory devices and the RS effect. Then, the recent progress of RS devices based on polymer materials, including single-component polymer materials, polymer composites, 2D covalent polymers, and biomacromolecules is summarized. Finally, we outline the challenges and outlook for the further development of polymer-based RS devices.

## 2. Overview of Polymer-Based RS Memory Devices

### 2.1. Device Structure

**Figure 1:**
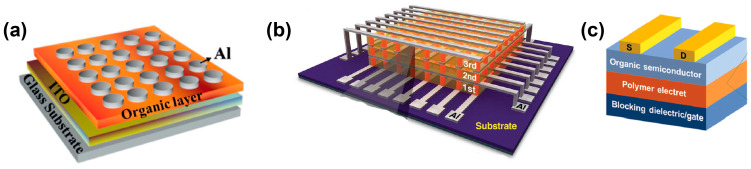
Schematic illustration of three typical RS memory device configurations. (**a**) Crossbar structure. Reproduced with permission [24]. Copyright 2021, Wiley-VCH GmbH. (**b**) Cross-array structure. Reproduced with permission [23]. Copyright 2010, Wiley-VCH GmbH. (**c**) Three-terminal FET RS device structure. Reproduced with permission [25]. Copyright 2019, The Royal Society of Chemistry.

Generally, there are two types of device structures, namely, the vertical metal–insulator–metal (MIM) structure and the lateral field-effect transistor (FET) structure in the polymer-based RS memory devices. These two structures are also called two-terminal and three-terminal devices, respectively (Figure 1). The electrodes are most widely made of Al, Au, Ag, Pt, p- or n-doped Si, indium tin oxide (ITO), or fluorine-doped tin oxide (FTO) [17]. Highly conductive reduced graphene oxide (rGO) has also been developed as an electrode material in addition to traditional metal electrodes. In a typical vertical MIM structure, the RS active layer is sandwiched between the bottom and top electrodes, which can be classified by crossbar and cross-array structures. These structures can provide massive device cell arrays, leading to high-density data storage. In particular, the cross-array geometry possesses more potential to scale down each device cell and realizes a highly integrated RRAM architecture. As a typical structure, Song et al. demonstrated a 3D-stacked crossbar arrayed RS device with a multilayer structure of Al/polyimide(PI):6-phenyl-C61 butyric acid methyl ester (PCBM)/Al/PI:PCBM/Al/PI:PCBM/Al. This structure presented a high storage density [23].

The three-terminal FET RS device is a transverse device structure with two laterally distributed electrodes and a semiconductor channel, as well as a gate electrode. The transverse configuration shows the advantage of being compatible with commercial complementary metal-oxide-semiconductor (CMOS) circuits. There are three types of FET RS devices, e.g., charge trap FET, floating-gate FET, and ferroelectric FET memory [26,27,28,29,30,31]. However, the operating voltage is relatively high in the FET RS device and this is a major issue that remains to be overcome. In this contribution, we mainly focused on the simple two-terminal MIM RS devices.

### 2.2. RS Memory Effect

**Figure 2:**
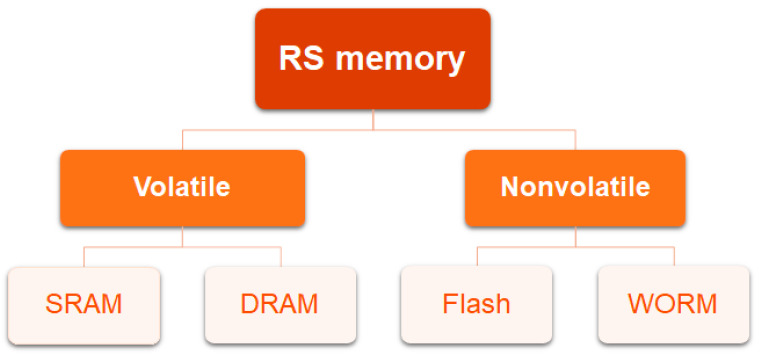
Schematic illustration of the RS memory types.

In the RS device, the writing operation is generated by applying a voltage bias or pulse to the device, leading to the conductance switching between the ON and OFF state. Depending on whether external electric power is required to maintain the ON state or not, the RS effect can be classified into volatile and non-volatile memory effects (Figure 2). The representative volatile memory types are the dynamic random-access memory (DRAM) and static random-access memory (SRAM) [32,33,34,35]. In DRAM, the ON state could be retained for a short period after turning off the applied voltage, while in SRAM, the device could sustain the ON state for a longer time than that observed in the DRAM device after the removal of the external power supply. Generally, in volatile memory, the ON state can be relaxed to the OFF state without an erasing process. This effect has potential for secure semiconductors and integrated electronic circuits.

In contrast to volatile memory, non-volatile memory can hold the stored information for quite a long time after the removal of the electric power. It can be divided into write-once-read-many-times (WORM) memory and rewritable flash memory [36,37,38]. In the former, the conductance switches from HRS to LRS under a certain voltage, and the LRS cannot be erased even if the external electric field is withdrawn. It is capable of maintaining the ON state permanently and shows extensive applications in rapid archival storage equipment, secure databases, as well as electronic labels. For the latter, the conductance switches between HRS and LRS. Flash memory is a promising candidate in data storage devices such as USB drives, hard disks, and other relevant rewritable digital storage.

### 2.3. Memory Mechanisms

**Figure 3:**
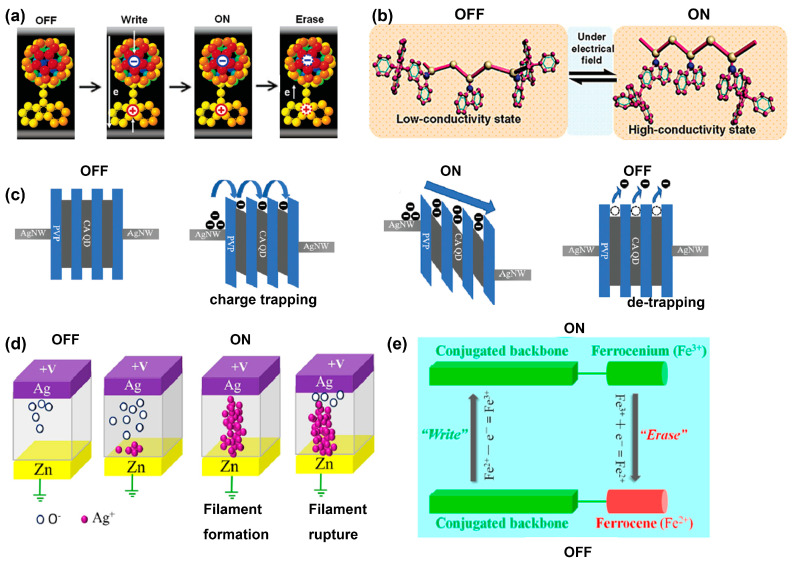
Schematic illustration of various switching mechanisms. (**a**) Charge transfer. Reproduced with permission [39]. Copyright 2007, American Chemical Society. (**b**) Conformational change. Reproduced with permission [40]. Copyright 2008, American Chemical Society. (**c**) Charge trapping/de-trapping. Reproduced with permission [41]. Copyright 2018, The Royal Society of Chemistry. (**d**) Filamentary conduction. Reproduced with permission [42]. Copyright 2018, American Institute of Physics. (**e**) Redox reaction. Reproduced with permission [43]. Copyright 2019, American Chemical Society.

Tremendous research has been devoted to elucidating the RS phenomena associated with polymer materials. However, the underlying mechanisms of the RS characteristics are still controversial. Based on the theoretical calculations and experimental analysis, some well-established memory mechanisms have been proposed, such as charge transfer, conformational change, charge trapping/de-trapping, filamentary conduction, and redox reaction, as shown in Figure 3.

#### 2.3.1. Charge Transfer

In a polymer with donor–acceptor (D–A) moieties, the charge transfer process usually occurs under an external electric field. The electronic charge will transfer from the donor to the acceptor moiety, leaving positively charged holes residing on the donors and the molecular orbitals partially filled. Hence, this process leads to the increase of the concentration of free charge carriers with high mobility, resulting in a high-conductive state (LRS). When an opposite electric field returns charges back to the donor group, the free charge carrier concentration will decrease and the D–A system will return back to HRS, showing the convertible charge transfer interaction. In order to obtain direct evidence of the charge transfer process, some theoretical calculations and experimental investigations have been conducted to show this process, e.g., density functional theory (DFT) calculations, UV–Visible absorption spectra, in situ fluorescence spectra and transmission electron microscope (TEM) images [39,44,45,46].

Interestingly, by tuning the dipole moment caused in the charge transfer process in D–A systems, volatile and non-volatile memory can be achieved. The strong dipole moment in polymers helps to sustain the high-conductive state, usually leading to non-volatile behavior. In contrast, a weak dipole moment leads to the unstable conductive state, and the volatile memory device will be realized [21].

#### 2.3.2. Conformational Change

The conformational change mechanism is mostly seen in polymers containing carbazole groups in the side chain such as poly(N-vinylcarbazole) (PVK) and its derivatives [40,47]. Initially, the random orientation of carbazole groups hinders the ordered π–π stacking and the charge transport is insufficient, indicating the HRS. With the external electric field, the carbazole groups are able to be rearranged into a nearly face-to-face π–π stacking, the ordered conformation can switch the polymers to LRS. In this process, excellent RS performances can be easily realized by the modification of polymer structures and conformational changes. The HRS to LRS transition is reversible by changing the electric field polarity, possibly due to thermal injecting at the electrode/polymer interface.

There are various characterization techniques to prove the conformational change of polymers, including DFT theoretical calculations, UV–Visible absorption spectra, in situ fluorescence spectra, X-ray diffraction (XRD), cyclic voltammetry (CV), TEM, and Raman spectra.

#### 2.3.3. Charge Trapping/De-Trapping

When metal nanoparticles, quantum dots, fullerenes, or organic semiconducting molecules are doped into polymer matrices, these dopants can act as trapping centers for charge carrier transport [41,48,49,50,51,52]. With increasing external voltage, charge carriers would be gradually injected into the trapping centers and local percolation networks will be formed. A continuous carrier hopping pathway will then switch the device from HRS to LRS.

By continually increasing the external electric field in the same polarity, the trapped charge carriers will exceed the capacity of the conductive channel and induce coulomb repulsion between the trapped charges to rupture the charge transport channel. Then, the device will switch back to HRS, behaving as unipolar RS memory. In contrast, when applying the reverse electric field to the device, the trapped charge carriers release from the trapping centers to rupture the conductive channel, leading to bipolar RS memory.

#### 2.3.4. Filamentary Conduction

Electrochemical metallization memory (ECM) and valence change memory (VCM) are the two representative types of filamentary conduction modes observed in some polymers for RS behavior [42,53,54,55]. By applying an electric voltage to the top electrode of active metals (e.g., Ag and Cu), the top electrode metal can be oxidated, and metal cations will be released from the top electrode to migrate through the active layer, which hence forms metal filament between top electrode and bottom electrode. The reverse voltage can deteriorate the conductive filament. Generally, VCM forms in donor-type defects such as mobile oxygen vacancies. Under an electric voltage, oxygen vacancies will be gathered at the cathode and diffused into the active layer to create a continuous conductive channel, which leads to a HRS to LRS transition. Under an opposite electric field, the conductive path is ruptured, which is known as a reset process [56].

The experimental methods to verify ECM and VCM mechanisms are relatively mature, e.g., the high-resolution TEM, scanning electron microscopy (SEM), and in situ scanning probe microscopy (SPM).

#### 2.3.5. Redox Reaction

In polymers with transition metal atoms, such as Fe, Co, or Mn in the backbone, the redox reactions of active materials are prone to occur between molecular reduction and oxidation states to alter the conductivity [43,57]. Unpaired or lone pair electrons can be removed, introducing impurity energy levels into the bandgap of active materials. These transition metal atoms usually possess various valences and can be switched between with the external voltage, resulting in binary or multilevel RS behavior. The positive charges can be balanced by reduction of environmental oxygen in the atmosphere or additional counter electrode materials, which may contribute to the stability of the redox system to enhance the endurance characteristics [58]. This electrochemical redox reaction phenomena are often certified by the CV technique to provide experimental evidence.

## 3. Polymer-Based RS Memory Devices

Polymer materials with the advantages of easy solution processability and intrinsic flexibility, are proving to be attractive for RS memory applications. Various facile low-cost solution methods, such as spin-coating, spray-coating, dip-coating, drop-casting, blade casting, and ink-jet printing are used to deposit polymer films. These materials used in RS memory devices can be classified into single-component polymers and polymer mixtures.

### 3.1. Single-Component Polymers

#### 3.1.1. Conjugated Polymers

Single-component polymers with donor–acceptor structures have potential charge transfer features, which are effective for realizing RS memories. The donor and acceptor moieties may be incorporated in the polymer backbone, linked as a side functional group, or dangling at the end of the macromolecular chain. By adjusting the strength and loading ratio, as well as spatial arrangement of the donor/acceptor moieties, volatile to non-volatile memory performance can be realized. To date, various conjugated and non-conjugated polymers have been reported to present RS properties.

**Figure 4:**
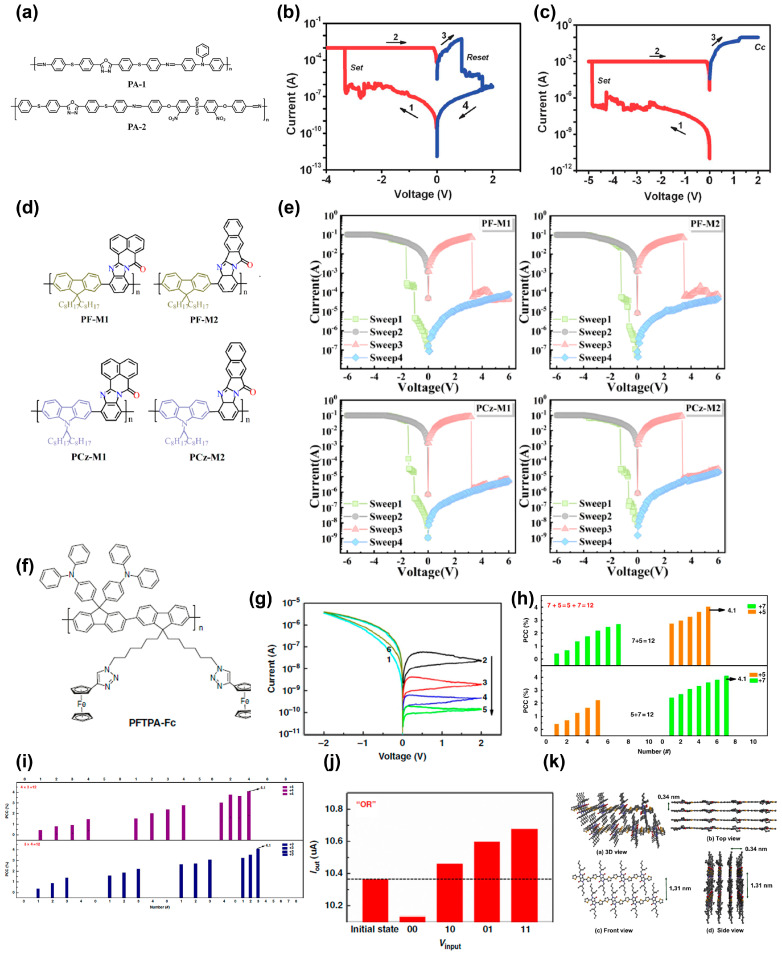
(**a**) Molecule structures of PA-1 and PA-2. *I–V* characteristics of the (**b**) Pt/PA-1/Pt and (**c**) Pt/PA-2/Pt devices. The arrows show the scanning direction of the applied voltage. Reproduced with permission [59]. Copyright 2013, The Royal Society of Chemistry. (**d**) Molecule structures of four donor−acceptor conjugated copolymers. (**e**) *I–V* characteristics of the memory devices based on the conjugated copolymers depicted. Reproduced with permission [60]. Copyright 2021, American Chemical Society. (**f**) Molecule structures of PFTPA–Fc. (**g**) *I–V* curves of the ITO/PFTPA–Fc/Pt device. The number 1-6 mean the six consecutive scanning of the voltage. (**h**) Demonstration of arithmetic commutative addition with the PFTPA–Fc memristor. (**i**) Demonstration of arithmetic multiplication with the PFTPA–Fc memristor. (**j**) Realization of the OR logic gate function with PFTPA–Fc memristor. The dotted line shows the initial device current of 10.36 μA. Reproduced with permission [61]. Copyright 2019, Springer Nature. (**k**) Schematic representations of molecular-chain conformation and packing structure in nanoscale poly(dtDPP) films. Reproduced with permission [62]. Copyright 2021, American Chemical Society.

The majority of RS memory polymers are based on π-conjugated nitrogen atoms, hydrocarbons, and their combinations. Polyazomethine (PAM) materials with imine groups (C=N) in the backbone are a typical type of conjugated polymers in RS memories. Li et al. synthesized two PAM derivatives, PA-1 and PA-2, as shown in Figure 4a [59]. In PA-1, the triphenylamine and oxadiazole moieties act as donor and acceptor, respectively. However, in PA-2, the oxadiazole acts as donor and the acceptor is 3,3′-dinitro-diphenylsulfone. With the device structure of Pt/active layer/Pt, PA-1 shows a rewritable RRAM memory effect with poor endurance, while PA-2 performs a WORM behavior (Figure 4b,c). Interestingly, by changing the bottom electrode to Al, both active layers exhibit the rewritable memory effect. For PA-1, the charge transfer interaction between the triphenylamine and the moderate electron withdrawing ability of oxadiazole is reversible, which results in rewritable memory behavior, whereas the charge transfer interaction between oxadiazole and 3,3′-dinitro-diphenylsulfone is rather strong, causing WORM switching behavior in PA-2. When the Al electrodes are introduced, the Schottky barrier at the Al–polymer interfaces become smaller with the presence of an ultrathin layer of Al_2_O_3_, which gives a lower reset voltage.

The majority of polymer-based memory devices present binary storage. In order to improve the capacity, Liu et al. synthesized the first single-polymer-based ternary memory device of iamP6 in 2012, combining the charge transfer and conformational change mechanisms [47]. Afterwards, interest in multilevel storage in a single polymer has been excited. The above research are the pioneering works on single-component polymer-based RS devices.

Recently, Zhang et al. connected naphthalene benzimidazole acceptor units to fluorene/carbazole donor, and four donor−acceptor conjugated copolymers were synthesized by the Suzuki reaction (Figure 4d) [60]. All of these polymers exhibit ternary electronic memory compared with pure fluorene/carbazole counterparts, as depicted in Figure 4e. After introducing monomers, two charge traps occur in the polymers. With increasing voltage to *V*_th1_, the injected charge carriers can transfer from the donor to the acceptor to fill the trap. Because the charge depth is associated with the acceptor group, resulting in different charge trap size, these traps cannot be filled simultaneously. The weak electron absorption ability of benzimidazole contributes to a small charge trap, which will be filled up at first, leading to the ON_1_ state. In contrast, the naphthalene structure has a stronger electron absorption ability with a large charge trap, which needs more energy to fill all charge traps to reach the ON_2_ state.

It is beneficial to incorporate redox active moieties onto the pendants of the polymers, to obtain astounding memory behavior. Zhang et al. introduced triphenylamine (TPA) and ferrocene (Fc) onto the sidechains of fluorene skeletons through the Suzuki reaction and “Click” chemistry, respectively, to achieve the final conjugated polymer PFTPA–Fc (Figure 4f) [61]. The ITO/PFTPA–Fc/Pt device exhibits four consecutive levels of RS characteristics by the electric-field-induced electrochemical reactions through three redox active moieties (Figure 4g). Moreover, four basic decimal arithmetic operations of addition, subtraction, multiplication, and division can be realized in the device (Figure 4h–j). This finding proves the feasibility of integrating multilevel memory and computing capability into a single memristive device by ingenious molecular design. Anchoring Fc in fluorene derivatives with porphyrin- and benzene-based diethynyl ligand, Roy et al. synthesized two metallopolymers (P1 and P2) [63]. Both polymers demonstrate WORM memory characteristics. Fc is effective in memristive polymer molecular design.

To achieve information storage and processing multifunctional memristors, Ren et al. introduced Ir complexes as electron-withdrawing groups on the polyfluorene backbone and synthesized a poly(9,9-dioctyl-9H-fluorene-*alt*-1,3-bis(2-ethylhexyl)-5,7-di(thiophen-2-yl)-4H,8H-benzo[1,2-c:4,5-c’]dithiophene-4,8-dione)-*alt*-(2,4-Pentanedionato)bis(2-(thiophen-2-yl)-pyridine)iridium) (PFTBDD-IrTPy) copolymer [64]. The synergetic electrochemical metallization and charge transfer effect between donors and acceptors are responsible for the memory behavior. The as-fabricated device can behave as an artificial synaptic emulation, simple Boolean logic, and decimal arithmetic calculation, which means it has so-called multibit data storage and processing capabilities in one memristor, like the findings reported in PFTPA–Fc.

Jung et al. designed several dithienyl-diketopyrrolopyrrole-based (dtDPP-based) narrow bandgap polymers, i.e., poly(2,5-bis(2-ethylhexyl)-3,6-di(thiophen-2-yl)-2,5-dihydropyrrolo[3,4-c]pyrrole-1,4-dione)) (poly(dtDPP)), poly(3-(5-(9,9-dioctyl-9H-fluoren-2-yl)thiophen-2-yl)-2,5-bis(2-ethylhexyl)-6-(thiophen-2-yl)-2,5-dihydropyrrolo[3,4-c]pyrrole-1,4-dione) (poly(dtDPP-FL)), and poly(3-(5-(4-(diphenylamino)phenyl)-thiophen-2-yl)-2,5-bis(2-ethylhexyl)-6-(thiophen-2-yl)-2,5-dihydropyrrolo[3,4-c]pyrrole-1,4-dione) (poly-(dtDPP-TPA)) [62]. Due to the self-assembled capability, poly(dtDPP) forms an edge-on layer structure, as can be seen from Figure 4k, whereas the others are preferentially oriented within the film. By adjusting the donor−acceptor powers in the backbone, poly(dtDPP) and poly-(dtDPP-TPA) show non-volatile WORM behavior, in comparison with poly(dtDPP-FL) exhibiting a DRAM behavior. Balancing of electron donor and acceptor powers is, in effect, adjusting memory type. Moreover, a redox active entity with thiophene-DPP donor and anthraquinone acceptor can also be used to obtain electrical bistability in the design of polymer memristive materials [65].

#### 3.1.2. Non-Conjugated Polymers

**Figure 5:**
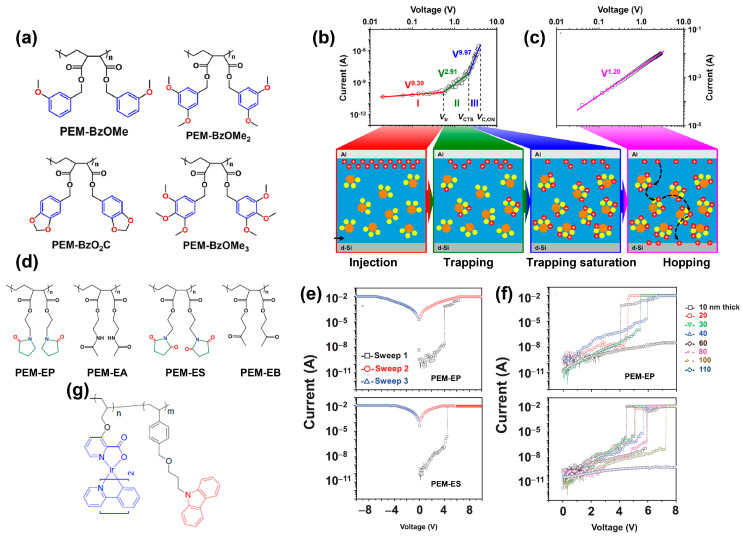
(**a**) Molecule structures of PEM-BzOMe, PEM-BzOMe_2_, PEM-BzO_2_C, and PEM-BzOMe_3_. (**b**) OFF-state of the measured *I–V* curves where the symbols are the measured data, and the solid lines represent the fit results using Schottky emission. Schematic diagrams of charge injection, trap, and trap-saturation are shown below. (**c**) ON-state of the measured *I–V* curves where the symbols are the measured data, and the solid lines represent the fit results using a hopping conduction model. Schematic diagrams of the hopping process are shown below. Reproduced with permission [66]. Copyright 2020, American Chemical Society. (**d**) Molecule structures of PEM-EP, PEM-EA, PEM-ES, and PEM-EB. (**e**) *I–V* characteristics of the memory devices based on PEM-EP and PEM-ES. (**f**) *I–V* curves of polymers of various thicknesses in sandwiched devices with a d-Si top electrode and an Al top electrode: PEM-EP (10–60 nm thick), and PEM-ES (10–110 nm thick). Reproduced with permission [67]. Copyright 2021, Wiley-VCH GmbH. (**g**) The chemical structures of iridium(III) complex as pendant groups. Reproduced with permission [68]. Copyright 2020, The Royal Society of Chemistry.

In addition to conjugated polymers, usually with a rigid backbone, non-conjugated polymers have the merits of environmental stability and flexibility and also enable RS behaviors. Beyond traditional hydrocarbons and nitrogen-based polymers, oxygen-containing electroactive polymers are superior for novel RS memory. In 2020, Ree et al. synthesized a series of poly(ethylene-*alt*-maleate)s derivatives with oxygen constituents and their derivatives as side groups through the postmodification reactions: poly(ethylenealt-di(3-methoxylbenzyl) maleate) (PEM-BzOMe), poly-(ethylene-*alt*-di(3,5-dimethoxylbenzyl) maleate) (PEM-BzOMe_2_), poly(ethylene-*alt*-dipiperonyl maleate) (PEM-BzO_2_C), and poly(ethylene-*alt*-di(3,4,5-trimethoxybenzyl) maleate) (PEM-BzOMe_3_), as depicted in Figure 5a [66]. The oxygen-containing polymers show superior thermal stability up to 180 °C, and exhibit reliable *p*-type unipolar volatile or non-volatile RS characteristics with high ON–OFF ratios ranging from 1.0 × 10^3^ to 1.0 × 10^8^. PEM-BzOMe and PEM-BzOMe_2_ exhibit excellent unipolar DRAM behavior in a limited thickness range. However, for PEM-BzO_2_C, WORM memory behavior can be observed in 17.7 nm devices and changes to DRAM memory mode with a film thickness less than <136 nm. A similar phenomenon also shows in PEM-BzOMe_3_. Figure 5b,c demonstrate the combination of Schottky emission and trap-limited SCLC conductions in the OFF-state and hopping conduction in the ON-state between charge trap sites are responsible for the memory behavior. This contribution proves that the RS behavior is controllable by tailoring the number of oxyphenyl units and/or oxy atomic components in the phenyl unit.

Ryu et al. first demonstrated 2-pyrrolidone and succinimide as electroactive elements in memory application [67]. Four polymers with and without 2-pyrrolidone and succinimide moieties were synthesized: poly(ethylene-*alt*-di(2-pyrrolidone-5-ethyl) maleate) (PEM-EP), poly(ethylene-*alt*-di(acetamidoethyl) maleate) (PEM-EA), poly(ethylene-*alt*-di(succinimido-N-ethyl) maleate) (PEM-ES), and poly(ethylene-alt-di(3-oxo-1-butyl) maleate) (PEM-EB). The chemical structures are presented in Figure 5d. It can be clearly seen from Figure 5e that PEM-EP and PEM-ES show non-volatile WORM memory over the film thickness range of 10–30 nm and 10–80 nm, respectively, whereas volatile digital memory can be seen in these two polymers over a rather narrower range of film thickness (Figure 5f). The succinimide moiety has relatively higher affinity and stabilization power, resulting in better memory performance.

By introducing iridium(III) complex as pendant groups in non-conjugated polymers, the model polymer is shown in Figure 5g. Yang et al. also realized volatile and non-volatile memory [68]. The concentrations of iridium(III) complex in the polymers affect the memory behavior. The polymers without iridium(III) complex shows SRAM behavior. The 4%, 8%, 12%, and 16% concentrations of iridium(III) complex in the polymers exhibit flash memory.

As a kind of polyanionic nano-cluster, polyoxometalate (POM) exhibits several discrete redox states in a narrow potential range, which can be used in multilevel memories. Hu et al. prepared a ternary redox POM-based inorganic–organic hybrid polymer, polymethyl methacrylate (PMMA)-MAPOM, by the copolymerization of MMA and MAPOM with 1,1′-Azobis(cyclohexanecarbonitrile) (ABCN) as the initiator [69]. The ITO/PMMA-MAPOM/Pt device presents rewriteable switching properties among three redox states under different reset voltages, showing multilevel properties and endurance over 50 cycles. The multi-redox states of manganese centers in polyoxoanion altering the effective carrier density in the switching layer is responsible for the switching behavior.

### 3.2. Polymer Mixtures

Polymers mixed with small molecules, nanoparticles, quantum dots, and hybrid perovskites to form polymer mixtures are widely reported in RS memory devices. They can integrate the merits of these materials to obtain high-performance memory devices.

**Figure 6:**
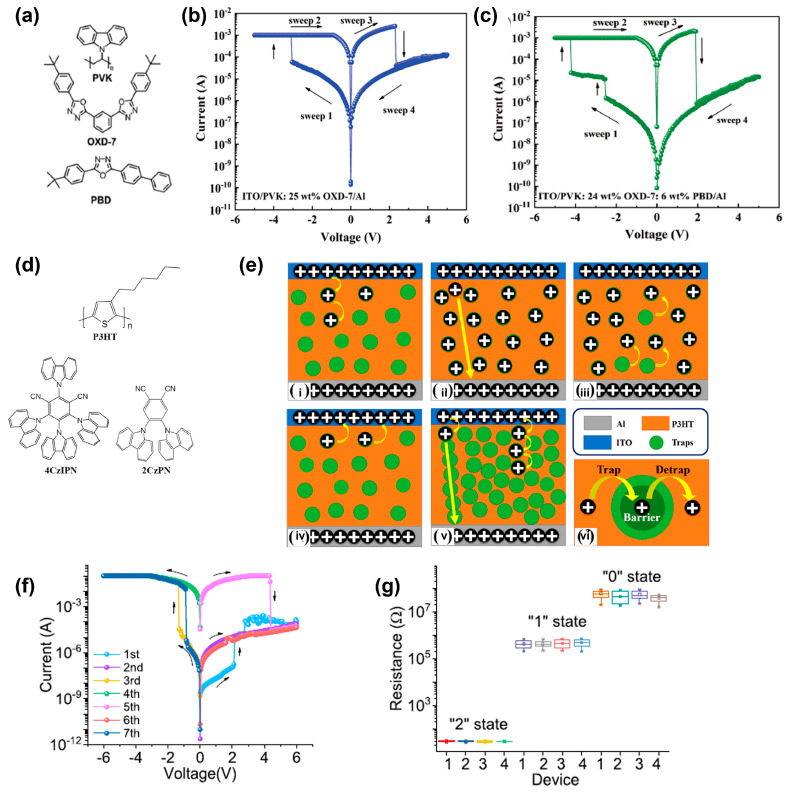
(**a**) Chemical structures of PVK, PBD, and OXD-7. (**b**) *I–V* characteristics of the ITO/PVK: 25 wt% OXD-7/Al devices. (**c**) *I–V* characteristics of the devices incorporated with PVK: 24 wt% OXD-7: 6 wt% PBD. Reproduced with permission [71]. Copyright 2021, The Royal Society of Chemistry. (**d**) Chemical structures of P3HT, 4CzIPN, and 2CzPN. (**e**) Schematic illustration of the switching mechanism. Charge transfer processes of (**i**) trap filling, (**ii**) fully filling trap, (**iii**) trap pumping, (**iv**) vacant trap, and (**v**) current leakage. (**vi**) Schematic illustration of the trap, de-trap, and charge barrier. Reproduced with permission [72]. Copyright 2022, American Chemical Society. (**f**) *I–V* curves of the fabricated multifunctional Al/PVP:thiadiazole/Al device with an initial positive voltage sweep. (**g**) Device-to-device “0”, “1”, and “2” tri-states variations in 109 cycles of four devices with initially applied positive voltage. Reproduced with permission [73]. Copyright 2019, Elsevier.

Polymers mixed with small molecular semiconductors can achieve the donor–acceptor structure, which is beneficial for memristive application. PVK and poly(3-hexylthiophene) (P3HT) are typical donors. The composite films of PVK mixed with carbon nanotubes (CNTs), and C_60_ have been reported [39,70]. The donor–acceptor system plays a vital role in binary RS devices. In particular, it is important to note that the donor–acceptor–acceptor system might be promising in ternary RS memristors. Pan et al. incorporated small molecular acceptors of 1,3-bis[2-(4-tert-butylphenyl)-1,3,4-oxadiazo-5-yl]benzene (OXD-7) into PVK to study the memory behavior, as illustrated in Figure 6a [71]. In Figure 6b, under 25 wt% of OXD-7, the composite films show stable memory curves with set and reset voltages of −3.1 and 2.3 V, respectively. Similar memristive behavior can also be seen with 30 wt% of OXD-7. When the concentration of OXD-7 increases to 40 wt%, no RS characteristic appears. They further introduced 2-(4-*tert*-butylphenyl)-5-(4-biphenylyl)-1,3,4-oxadiazole (PBD) to construct the donor–acceptor–acceptor system. The composite film of PVK (24 wt% OXD-7:6 wt% PBD) exhibits remarkable ternary RS behavior with a switching ratio of 1:10:10^4^ in Figure 6c. The ON–OFF ratios in the donor–acceptor–acceptor systems are generally higher than those in donor–acceptor systems. The electric-field-induced charge transfer between PVK donor and oxadiazole moiety-formed OXD-7 and PBD is responsible for the RS effect. Li et al. reported memristive behavior based on P3HT mixed with 2,4,5,6-tetrakis(carbazol-9-yl)-1,3-dicyanobenzene (4CzIPN) or 4,5-bis(carbazol-9-yl)-1,2-dicyanobenzene (2CzPN) composites (Figure 6d) [72]. The two carbazolyl dicyanobenzenes with low intrinsic mobility and high steric hindrance might inhibit the leakage current of the HRS. Dramatically, these composite films show switching ratios higher than 10^5^, retention times of more than 5 × 10^4^ s, and endurance cycles of 150 times. The charge trapping and detrapping process leads to the charge transport channels, which are responsible for the memory behavior. The memory mechanism is comprehensively illustrated in Figure 6e. This finding reveals the effect of intermolecular interaction on RS behavior. Sun et al. implanted 2-Amino-5-methyl-1,3,4-thiadiazole into poly(4-vinylphenol) (PVP) to construct a Al/PVP:thiadiazole/Al device [73]. Both non-volatile WORM and flash memory behaviors are present in a single device. From Figure 6f, in the forward voltage sweep, the device first shows WORM behavior. Then, in the reverse voltage sweep, the device shows a second “write” operation and the “erasing” operation appears in the following forward scanning direction. Therefore, the device could realize “0”–“1”–“2”–“1”–“2” tri-stable resistance states and the device-to-device variations and tri-state variations in 109 cycles are presented in Figure 6g. The “0” state is not the electroforming state, resulting in an electroforming-free device. This work provides a new strategy for designing ternary data storage utilities.

**Figure 7:**
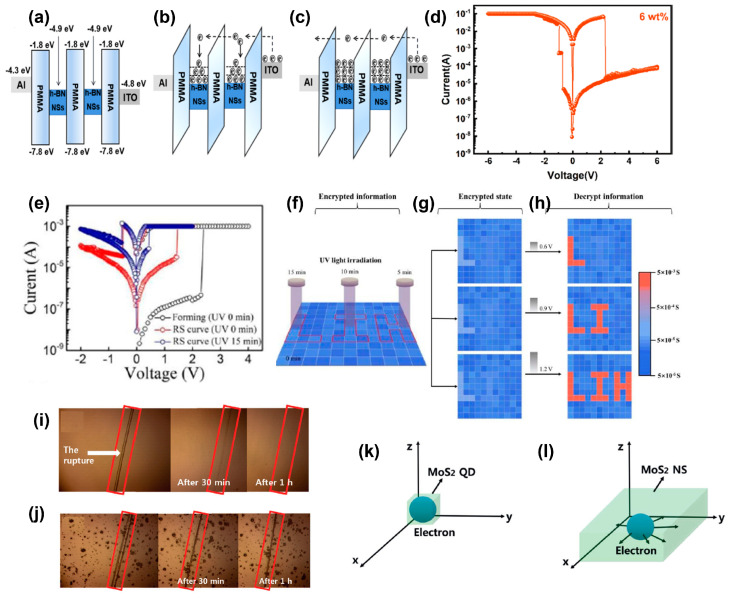
(**a**) The energy band diagram for the Al/h-BN NSs:PMMA/ITO/PEN devices and the carrier transport processes of (**b**) HRS, and (**c**) LRS. Reproduced with permission [74]. Copyright 2021, Elsevier. (**d**) *I−V* curves of PIIO:6 wt% WS_2_ nanoparticles. Reproduced with permission [75]. Copyright 2022, American Chemical Society. (**e**) The effects of UV irradiation on RS behaviors of an ITO/PVP-NCQDs/Al memory device. (**f**) The UV irradiation represented the process of information encryption, in which three regions (the image “L”, “I”, and “H”) underwent 15, 10, and 5 min UV irradiation, respectively. (**g**) Read pulses with different amplitudes (0.6, 0.9, 1.2 V) were applied to the encrypted state. (**h**) Diverse images can be decrypted including image “L”, image “LI”, and image “LIH”, respectively. Reproduced with permission [76]. Copyright 2020, The Royal Society of Chemistry. (**i**) PVA-IMGQD film heated at 50 °C for 1 h showed almost complete healing. (**j**) PVA-pure GQD film heated at 50 °C for 1 h showed almost no healing. Reproduced with permission [77]. Copyright 2021, The Royal Society of Chemistry. Wiley-VCH GmbH. Schematic diagrams of electrons confined in (**k**) MoS_2_ quantum dots and (**l**) MoS_2_ NSs. Reproduced with permission [78]. Copyright 2021, Wiley-VCH GmbH.

Polymers could also act as matrixes for nanoparticles or quantum dots for memory operations. Nanoparticles usually act as a trap center in the switching layer. Kim et al. reported a flexible and stable memristive devices consisting of hexagonal boron nitride nanosheets (h-BN NSs):PMMA nanocomposites [74]. H-BN has a smooth atomic surface, no dangling bonds and excellent thermal stability. The strong electron binding force of h-BN makes it suitable for use as a carrier trapping center in the memory. The device shows a WORM character with an ON–OFF ratio of 10^3^. The flexible device on polyethylene naphthalate (PEN) substrate can maintain the memory properties over 2 × 10^3^ bending cycles. The discrete energy level state causes a strong quantum confinement effect in the h-BN NSs, as shown in Figure 7a–c, which is responsible for the WORM effect. Zhou et al. investigated WS_2_ nanoparticles, which have a large specific surface area and good conductivity, to be doped in poly[2,7-9-(9-heptadecanyl)-9*H*-carbazole-*co*-benzo[4,5] imidazole[2,1-α] isoindol-11-one] (PIIO) matrix [75]. The incorporation of 6 wt% WS_2_ nanoparticles showed the best non-volatile ternary storage features with the switching ratios of 1:1.11 × 10^1^:2.03 × 10^4^ for three resistance states (Figure 7d). The WS_2_ nanoparticles lower the charge injection barrier and induce conductive pathways and conductive filaments.

Carbon quantum dots with small size, high electron transfer efficiency, and attractive optical properties are promising in optoelectronic applications. Lin et al. investigated a memory device based on PVP and N-doped carbon quantum dot nanocomposites to observe photo-tunable memory behavior [76]. The set voltages and the switching ratios decrease with the increasing time of UV light irradiation (Figure 7e). UV light induces local conductive amorphous carbon region, which can enhance the internal electrical field and shorten the charge tunneling channel. As shown in Figure 7f–h, with a 9 × 9 RRAM array, this device can realize encrypted image storage. The inputted images of letters “L”, “I”, and “H” were irradiated by UV light for 15, 10, and 5 min, respectively. Herein, they can be readout through electric voltage pulses with different amplitudes on the encrypted RRAM array. Jiang et al. reported a carbon dot:PVP nanocomposite-based RS device with silver nanowires as top and bottom electrodes on the flexible gelatin film substrate [79]. The memory behavior shows negligible fluctuations over 100 bending cycles. The trap-related SCLC is attributed to the RS mechanism. The all-biocompatible materials exhibit excellent degradability. The device can dissolve completely within 90 s after being submerged in deionized water at 55 °C and degrade naturally in soil within 6 days. This work paves the way for carbon dots in flexible and wearable green electronics.

Wearable electronic devices may be damaged under repetitious mechanical stress. Kim’s group presented a self-healable RS device based on a composite layer composed of a PVA matrix and imidazole-modified graphene quantum dots (IMGQD) [77]. The device exhibits WORM behavior with a set voltage of 1.7 V. Figure 7i,j depict that the PVA–IMGQD films can be completely self-healed after 1 h at 50 °C. Meanwhile, the PVA film doped with pure GQDs does not show self-healing. The imidazole groups in the IMGQDs are the key factor to obtain self-healing. At the crack interface of the films, the PVA–IMGQD chains on both sides are gradually recombined due to hydrogen bonding, leading to self-healing. When the device is completely cut off, including the ITO bottom electrode, the current will gradually recover with the progress of self-healing. At the same time, the retention and durability of the device are nearly unchanged. This excellent result is of great importance for the development of portable electronic systems.

To enhance the thermal stability of the memristive device, molybdenumdisulfide (MoS_2_) quantum dots with high temperature resistance and strong quantum confinement effect were incorporated into the polyimide (PI) matrix [78]. The ITO/PEDOT:PSS/PI-MoS_2_ quantum dot/Al structure exhibits WORM characteristics in the voltage range from −6 to 3 V. However, the PI–MoS_2_ nanosheet (NS)-based devices show bipolar flash memory. The WORM behavior is attributed to the strong binding force of the quantum confinement effect in the doped MoS_2_ quantum dots. The electrons are trapped in three dimensional directions in the quantum dots, while for the nanosheets, the electrons are only limited in the *z* direction and they can be released under a certain reverse voltage (Figure 7k,l). No significant degradation of the memory characteristics can be observed under high annealing temperatures of 50, 100, and 200 °C, showing the high thermal stability of MoS_2_ quantum dot.

Chalcogenide-based colloidal CdSe quantum dots were embedded in PVP matrix to enhance the switching ratio, which was reported by Sahu et al [80]. They found a nanoscale heterostructure formed with colloidal monodispersed CdSe quantum dots and PVP, which helps to achieve the high ON–OFF ratio of 10^5^. The memory mechanism is analyzed from fitting the *I–V* curves. The charge conduction in the HRS state is due to hot-charge-injection and space-charge-injection conduction. This switches to Ohmic conduction in the LRS state.

**Figure 8:**
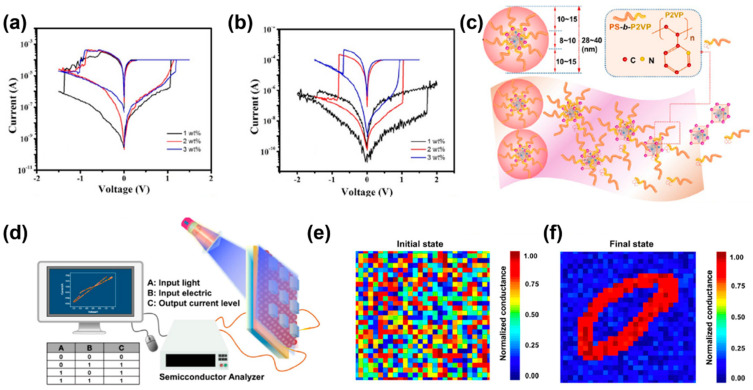
*I–V* curves of the composite devices in various concentrations for (**a**) an ITO/Cs_2_AgBiBr_6_@PEO/Au device, and (**b**) an ITO/Cs_2_AgBiBr_6_@PMMA/Au device. Reproduced with permission [88]. Copyright 2023, Elsevier. (**c**) Schematic illustration of S2VP:CsPbBr_3_ quantum dots core-shell nanosphere composite. (**d**) Schematic illustration of the S2VP−CsPbBr_3_ quantum-dot-based logic OR device. Mapping a representative input digit of 784 synaptic weights connected to the output digit “0” shown at the (**e**) initial and (**f**) final states of training. Reproduced with permission [89]. Copyright 2023, American Chemical Society.

Nowadays, organic–inorganic hybrid perovskites (OIHPs) have attracted increasing attention in optoelectronic devices due to their high linear absorption coefficient, tunable bandgap, long exciton diffusion length, high electron mobility, high crystallinity, and solution processability [81,82,83]. Great efforts have been devoted to OIHP-based RS memory devices [84,85,86]. Pristine perovskite or perovskite quantum dots can also be utilized in a polymer matrix to improve the memristive behavior. In 2016, Chen et al. pioneered the use of CH_3_NH_3_PbI_3_:PVK for blending active layers to construct a bulk heterojunction (BHJ) concept as popularly used in solar cells [87]. This BHJ-based device exhibits a non-volatile WORM memory with a large ON–OFF ratio of more than 10^3^. PVK and CH_3_NH_3_PbI_3_ act as a donor and acceptor, respectively. The intermolecular charge transfer between the donor and the acceptor induced by the electric field has been attributed to the WORM properties. Polymer matrix may influence the polymer–perovskite composite-based memory behaviors. Zhang et al. compared the PMMA and polyethylene oxide (PEO) matrixes with respect to the RS performance of Cs_2_AgBiBr_6_ double-perovskite nanofillers [88]. The PEO-based device does not depict significant changes in RS performance upon Cs_2_AgBiBr_6_ doping. However, an obvious impact of 2 wt% Cs_2_AgBiBr_6_ in PMMA-based devices could be achieved with low set and reset voltages, and a high ON–OFF ratio of 10^4^ (Figure 8a,b). The pristine PEO has higher ionic conductivity than that of PMMA, which is nearly an insulator. Herein, a higher HRS current caused by ionic conductivity could be observed in a PEO-based device. The conductivity of the polymer matrix has a crucial effect on RS performance.

CsPbBr_3_ quantum dots are easy to decompose with environmental water and oxygen. Very recently, Jiang et al. used an amphiphilic diblock copolymer polystyrene-poly2-vinyl pyridine (PS-*b*-P2VP, S2VP) to protect the CsPbBr_3_ quantum dots and realized a core–shell nanosphere composite (Figure 8c), leading to a robust and light-tunable memristor [89]. The device demonstrates ultra-stable RS behavior over 5000 cycles and over 5 million seconds, rendering it favorable for light tunability. Light and external electric fields can effectively change the resistance state of the device to realize the logical operation (Figure 8d). This light-tunable behavior can be used in biologically visually inspired neuromorphic computing. Simple machine learning was illustrated by simulating optoelectronic neural network learning. They used a single-layer perceptron model to categorize 28 × 28 pixels of handwritten digital images from a National Institute of Standards and Technology (MNIST) dataset using a backpropagation algorithm to perform supervised learning, as shown in Figure 8e,f. After 900 training epochs, the device demonstrates a maximum recognition accuracy of 97%, higher than the previous reports.

## 4. 2D Covalent Polymer-Based RS Memory Devices

Different from traditional organic polymers, 2D covalent polymers are synthesized through covalent bonding of pre-designed molecular building blocks to form covalently linked networks of monomers with periodic structures in two orthogonal directions [90]. They have been successfully prepared by the Langmuir–Blodgett (LB) method, chemical vapor deposition (CVD), and surface confined synthesis [91,92,93]. Covalent bonds are very stable, rendering these polymers with high stability in many solvents and harsh environments. Additionally, 2D polymers (2DPs) are stable in monolayers with sub-nanometer thickness, which is similar to graphene. Hence, the monolayer 2DPs are expected to reduce the filament length and minimize the energy to form and rupture the conductive filaments. These unique properties of 2DPs make them promising candidates for the advanced RS memories [94].

**Figure 9:**
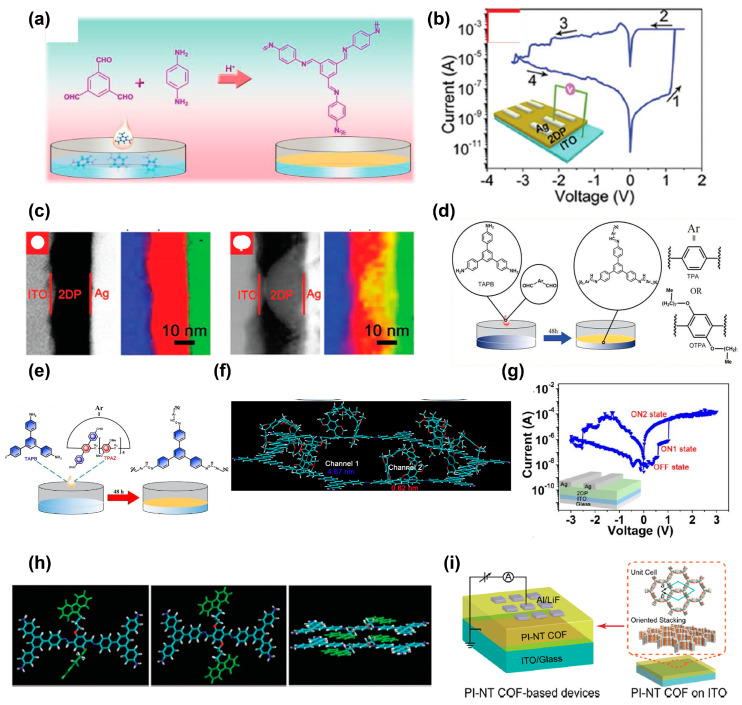
(**a**) A schematic illustration of the synthesis of 2DP_BTA+PDA_ films through the Schiff-base reaction of the monomers. (**b**) *I–V* curves for the ITO/2DP_BTA+PDA_/Ag configuration. (**c**) From left to right: ADF STEM image and chemical maps of ITO/2DP_BTA+PDA_/Ag device in the initial and on state. Reproduced with permission [95]. Copyright 2019, WILEY-VCH. (**d**) The preparation diagram of MP_TPA+TAPB_, MP_OTPA+TAPB_ on the solution/air interface. Reproduced with permission [96]. Copyright 2020, The Royal Society of Chemistry. (**e**) The preparation diagram of 2DP_TPAZ+TAPB_ at the solution/air interface. (**f**) Simulated structure of the 2DP_TPAZ+TAPB_ material part unit. (**g**) *I–V* characteristics of the ITO/2DP_TPAZ+TAPB_/Ag device. Reproduced with permission [97]. Copyright 2021, WILEY-VCH. (**h**) The conformation change memory mechanism. Reproduced with permission [98]. Copyright 2022, The Royal Society of Chemistry. (**i**) Schematic diagram of the PI–NT COF film stacking and the ITO/PI-NT COF film/LiF/Al configuration. Reproduced with permission [99]. Copyright 2020, American Chemical Society.

In 2019, Liu et al. first investigated an innovative 2DP-based non-volatile memristive device [95]. A wafer-scale ultrathin 2D imine polymer film was synthesized through a Schiff base polycondensation reaction from benzene-1,3,5-tricarbaldehyde (BTA) and *p*-phenylenediamine (PDA) building blocks (Figure 9a). Notably, the construction of a memory device based on 2DP_BTA+PDA_ films presents superior RS behaviors with an ON–OFF ratio from 10^2^ to 10^5^, an impressive retention time of 8 × 10^4^ s and a stable endurance of 200 cycles (Figure 9b). The superior thermal stability allows the 2DP_BTA+PDA_-based device to show an increasing ON–OFF ratio with increasing annealing temperature at 300 °C, and this non-volatile memory behavior is also stable in polar and non-polar solvents. A flexible device with graphene/2DP_BTA+PDA_/Ag structure on a polyimide (PI) substrate exhibits excellent memory behavior and mechanical durability over 500 bending cycles. Good flexibility and thermal stability give 2DP_BTA+PDA_ great potential in wearable electronics. The Ag conductive filament resistances are responsible for the switching mechanism of this device, which is certified by annular dark-field scanning transmission electron microscopy (ADF STEM) and electron energy loss spectrum (EELS), as shown in Figure 9c. These findings indicate the promising potential of 2DP thin films in next-generation memory devices and lead to the development of reliable memory devices.

Later, to demonstrate the effect of chemical structures of microporous polymers (MP) on the performance of memristor, they continued to synthesize two microporous covalent polymers (MP_TPA+TAPB_, MP_OTPA+TAPB_) with tris(4-aminophenyl)-benzene (TAPB) to react with terephthalaldehyde (TPA) and 2,5-dioctyloxyterephthalaldehyde (OTPA), as schemed in Figure 9d [96]. The incorporation of octyloxy groups within the dialdehyde monomer reduces the band gap and changes the pore environment. Thereafter, the HRS resistance is reduced, resulting in an increase in the ON–OFF ratio by an order of magnitude. The switching mechanism is attributed to the electrochemical metallized Ag conductive filaments that connect the top and bottom electrodes. Their findings provide a design criterion between molecular structures and memory properties.

Hu’s group further explored the ternary electronic memory of 2DP with 2,5-bis(3-(9H-carbazol-9-yl)propoxy)terephthalaldehyde (TPAZ) and TAPB as monomers in 2021 (Figure 9e) [97]. The intrinsic subnanometer channel of pillar [5] arene and nanometer channel of 2DP construct multilevel channels for ternary memory devices based on 2DP_TPAZ+TAPB_ (Figure 9f). The device exhibits a high ON–OFF ratio of 1:10:10^3^, and a high ternary yield of 75%, as shown in Figure 9g. The 2DP_TPAZ+TAPB_ also presents stable flexible ternary memory after bending for 500 cycles, as well as thermal stability to a high temperature of 300 °C.

The memory mechanisms of the above research are mainly based on conductive filaments. Hu’s group integrated the conformational change mechanism in a 2DP reaction between 2,5-bis(3-(9H-carbazol-9-yl)propoxy)terephthalaldehyde (TPAK) and TAPB [98]. By controlling the compliance current (*I*_CC_), three RS memory behaviors, including non-volatile WORM, non-volatile FLASH memory, and volatile dynamic DRAM behavior, were achieved with the configuration of ITO/2DP_TPAK+TAPB_/Au. Specifically, the devices display volatile DRAM at *I*_CC_ = 10^−4^ A, while they show non-volatile FLASH and WORM memory behaviors at *I*_CC_ = 10^−3^ and *I*_CC_ = 10^−1^ A, respectively. Figure 9h shows the conformation change in the carbazole groups through their rotation, leading to more regular π–π stacking, which is confirmed by UV–Visible spectra.

In 2023, Hu’s group demonstrated highly crystalline single-layer 2D polymers (SL-2DPs) via the condensation of TAPB and terephthalaldehyde decorated with different lengths of alkoxy chains (TPOC_x_, x = 0, 2, 4, 8, 12, 16, 22) using a LB method [100]. The long alkoxy chains were incorporated to enhance the crystalline structure of 2DP. The devices based on SL-2DPs show an ultralow working voltage (0.6 V), good endurance (324 cycles), and long retention times (10^5^ s). Additionally, the device exhibits excellent mechanical flexibility and electrical reliability. The memory behavior is still maintained under the strains of 2.6%. The research by Hu’s group suggests the potential for 2DP in emerging applications such as dense data storage and ultra-thin, highly stable, flexible electronics.

The 2DPs mentioned above typically exhibit a π-conjugated structure. However, incorporating a donor–acceptor moiety into 2DPs has been shown to result in more effective and durable RS memory behaviors. In 2020, Sun et al. demonstrated the first donor–acceptor two-dimensional polyimide covalent organic framework (2D PI–NT COF) films with 4,4′,4″-triaminotriphenylamine (TAPA) and naphthalene-1,4,5,8-tetracarboxylic dianhydride (NTCDA) as donor and acceptor, respectively (Figure 9i) [99]. The high-quality film exhibits high crystallinity, good orientation, and tunable thickness. The memory device shows superior WORM performance, with an ON–OFF ratio exceeding 10^6^, a retention time of 10^4^ s, and high stability. Due to the donor–acceptor moieties, an electric-field-induced intramolecular charge transfer is attributed to the memory behavior.

Li and coworkers further introduced an electron acceptor, [2,2′-bithiophene]-5,5′-dicarbaldehyde (BTDD) or (*E*)-5,5′-(ethene-1,2-diyl)bis(thiophene-2-carbaldehyde) (TVTDD), and an electron donor, 4,4′,4″-(1,3,5-triazine-2,4,6-triyl)trianiline (TAPT), into 2D imine frameworks to achieve COF–TT–BT and COF–TT–TVT [101]. The 100 nm thick COF–TT–BT and COFTT–TVT films show rewritable memories with high ON–OFF ratios (10^5^ and 10^4^) and low driving voltages (1.30 and 1.60 V), which are different from the WORM of 2D PI–NT COF. The energy level alignment between the COF and the electrodes may impact the mechanism of RS. Chen’s group developed COF-based redox activity memory materials, namely COF–Azu, which is made by combining TAPB, azulene 1,3-dicarbaldehyde (Azu), and TFPB–PDAN by the co-condensation of 1,3,5-tris(4-formylphenyl)-benzene (TFPB) and *p*-phenylenediacetonitrile (PDAN) [102,103]. The electric-field-induced charge transfer in the donor–acceptor structure is responsible for the memory. These results further fulfill the application of 2D COF materials in high-performance RS memory devices.

The 2D structural homogeneity may promote effective charge transport and improve the performance of the polymer-based memory device. To overcome the low production yield and reliability of the device, Zhang et al. designed a 2D conjugated polymer, PBDTT–BQTPA, composed of redox active triphenylamine moieties anchored on the coplanar bis(thiophene)-4,8-dihydrobenzo[1,2-b:4,5-b]dithiophene (BDTT) donor and quinoxaline acceptor [104]. The coplanar structure and ordered π–π stacking of the macromolecule backbone render the polymer with enhanced crystalline uniformity; therefore, the production yield rises to 90%, with switching parameter variation decreasing to 3.16–8.29% and potential scalability changing into a 100 nm scale.

## 5. Biomacromolecules-Based RS Memory Devices

Biomacromolecules can be easily obtained by extraction from living organisms, without needing a complex chemical synthesis route. With the advantages of biocompatibility, environmental friendliness, and flexibility, the electronic devices based on biomaterials are generally cost-effective, ecofriendly, and sustainable. Biomaterials show interesting applications in implantable biomedical devices. In addition, their self-decomposition behaviors make them valuable for applications related to security circumstances. Various studies have been devoted to biomaterial-based RS memory devices, including chitosan, starch, lignin, protein, glucose, enzyme, and DNA [105,106].

**Figure 10:**
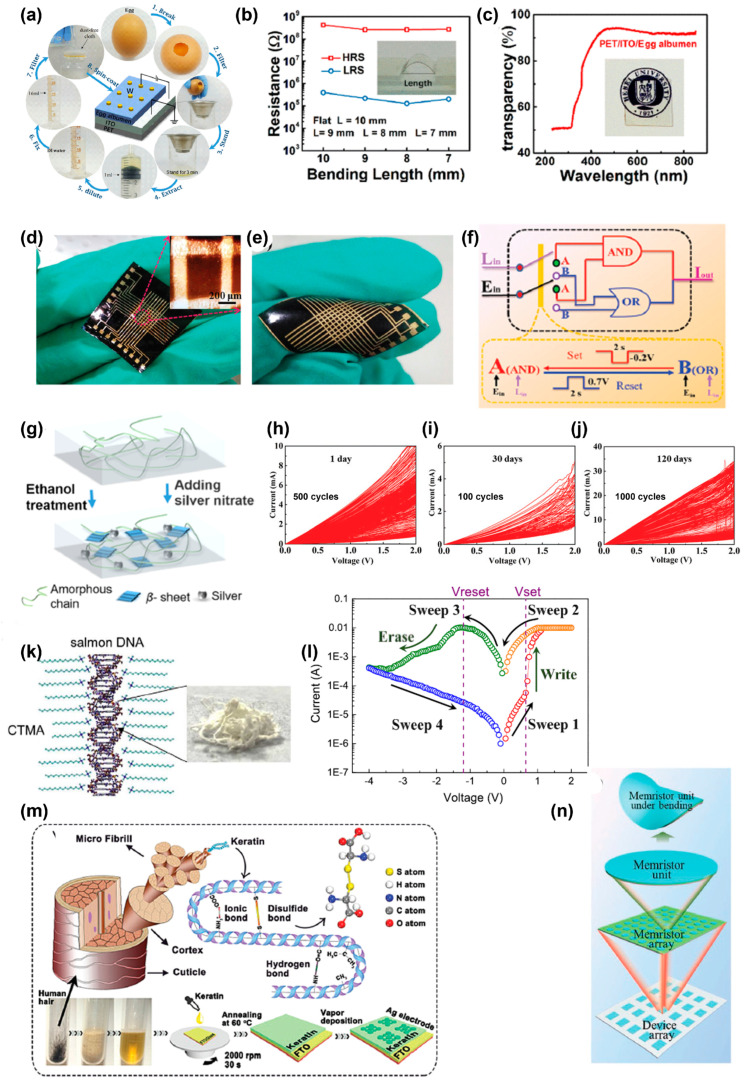
(**a**) Proposed fabrication process and schematic of a PET/ITO/egg albumen/W memristor. (**b**) The device’s HRS and LRS versus bending length. (**c**) Transparency demonstration of the device. Reproduced with permission [107]. Copyright 2019, American Chemical Society. Photographs of the Au/albumen/Au crossbar arrays in (**d**) flat, and (**e**) moderate-angle bending states. (**f**) A schematic diagram of the memory logic gate switching between ”OR” and ”AND” states. Reproduced with permission [108]. Copyright 2019, The Royal Society of Chemistry. (**g**) Schematic of the conformational transition of silk fibroin with Ag doping and ethanol treatment. Reproduced with permission [111]. Copyright 2021, American Chemical Society. (**h**–**j**) The endurance and retention test for the analogue RS memristor after: 1 day; 30 days; and 120 days. Reproduced with permission [112]. Copyright 2021, The Royal Society of Chemistry. (**k**) The chemical structure of DNA–CTMA. (**l**) *I–V* curve of the fabricated device based on DNA–CTMA. Reproduced with permission [114]. Copyright 2018, Elsevier. (**m**) Schematic diagrams of keratin from human hair, the chemical bonds and structures in keratin, and the fabrication process of FTO/keratin/Ag memory devices. Reproduced with permission [115]. Copyright 2019, The Royal Society of Chemistry. (**n**) Schematic diagram of a device with a crossbar array and a cell being bent. Reproduced with permission [116]. Copyright 2023, Wiley-VCH.

As a common biomaterial in our daily life, egg albumen exhibits promising memristive behavior in transparent and flexible memristor devices. A flexible polyethylene terephthalate (PET)/ITO/egg albumen/tungsten device (Figure 10a), shows outstanding memristive operation under mechanical bending without significant performance degradation and possesses a transparency of more than 90% with visible light in a wavelength range of 230−850 nm (Figure 10b,c) [107]. This device can mimic certain neural bionic behaviors to present typical synapse performance, i.e., short-term plasticity (STP), long-term plasticity (LTP), and transitions between STP and LTP. Additionally, it can be dissolved in deionized water within 1 day. These results show that albumen-based devices are attractive as biocompatible and biodegradable electronics. As shown in Figure 10d,e, Zhou et al. fabricated albumen-based large-area paper substrates exhibiting both robust physical flexibility and excellent electric properties [108]. The crossbar arrays show an ON–OFF ratio of 10^4^, and a retention time of 10^4^ s even after bending 10^4^ times. This device can realize complete memory logic blocks containing NOT, OR, and AND gates (Figure 10f). Wang et al. further explored egg albumen’s application in a logic circuit [109]. With the configuration of Al/PMMA/egg albumen:Au nanoparticles/PMMA/Al, the ON–OFF ratio is enhanced dramatically, to 2.86 × 10^5^, compared with the device without PMMA as insulating layer. The oxygen-vacancy-dominated conductive filaments are responsible for the RS mechanism. The device also presents full basic logic functions, including AND, OR, NOT, NAND, and NOR, based on auxiliary logic. By introducing multiwalled carbon nanotubes (MWCNTs) into egg albumen, the switching ON–OFF ratio of ITO/egg albumen:MWCNTs/Al device increases as the concentration of MWCNTs decreases [110]. By controlling the compliance current, the multilevel RS memory realizes 2-bit data storage to increase the storage density. These egg-albumen-based functional materials meet most of the required standards of electrical characteristics for an RRAM including a high ON–OFF ratio, high electrical endurance, long retention time, fast switching speed, and low power consumption. Therefore, it plays a pivotal role in the non-volatile memory device.

Silk fibroin is derived from natural silk. In order to improve the stability and power consumption of silk-fibroin-based memristors, Zhang et al. doped the silk fibroin with Ag and an ethanol-based post-treatment to form microcrystal regions in the bulk of the silk fibroin, as can be seen in Figure 10g [111]. The microcrystal regions make the charge carriers transport through the fixed and short paths, resulting in a high stability and low power consumption (0.7 μW) memristor. The switching mechanism is attributed to the SCLC mechanism. The non-linear transmission function of synapses shows the great potential of silk fibroin in artificial synapses. Wang et al. synthesized a novel protein-based polymer by the polymerization of silk fibroin and 2-isocyanatoethyl methacrylate [112]. The polymer acquired an analogue-type computing behavior characterized by more than 32 programmable conductance states. The analogue property does not show any depression in ambient air for 4 months (Figure 10h–j). A physical model consisting of the trapping and de-trapping of the injected electrons may be responsible for the analogue-type behavior. The electrodes play a vital role in memory behavior. With a GO/silk fibroin/GO structure, Liu et al. reported multilevel storage with binary and ternary switching behaviors in a single device [113]. For *I*_cc_ ≤ 0.01 A, the device exhibits binary switching because of the SCLC mechanism, while *I*_cc_ exceeds 0.01 A, the device changes to ternary switching behavior, due to the SCLC and Poole–Frenkel emission mechanisms. By analyzing handwritten numbers obtained from the Modified National Institute of Standards and Technology database, this memristor-based ternary weight network exhibits a recognition accuracy of 92.3%, which is better than that based on a binary neural network. This study shows the application of silk fibroin in neural morphological computing.

DNA molecules are complete in nature and can be easily extracted from biological species. Since Hung et al. pioneered the investigation into the DNA-based transparent WORM device, the DNA in RS memory have been comprehensively explored [117]. Jeng et al. fabricated a DNA-based RRAM device with DNA–cetyltrimethylammonium (CTMA) macromolecules (Figure 10k) [114]. The ITO/DNA–CTMA/Ag device shows low set and reset voltages of 0.65 V and −1.25 V, respectively. Figure 10l shows that it has an ON–OFF ratio of 10^2^, an electrical endurance of 200 cycles, and a long retention time of 10^4^ s. Notably, the device exhibits a pronounced negative differential resistance (NDR) region. Multilevel resistances can be observed by adjusting the magnitude of negative biases. The conduction mechanism was due to the formation of a conductive Ag filament, which was ascertained from completely studying the electrical properties at different temperatures, active layer thicknesses, and electrode materials. This finding paves the way toward future integration of natural DNA-based biomaterials. Abbass et al. fabricated a transparent memory device by sandwiching a Cu^2+^-doped salmon DNA molecule between FTO and Pt [118]. The device shows the set and reset voltages of −3.5 and 2.5 V, respectively. Unlike the majority of RRAM devices, the Cu^2+^-doped device shows LRS initially with an ON–OFF ratio of 10^3^, a retention time of 10^4^ s, and an endurance of 10^5^ pulses. The migration of Cu^2+^ under externally electric power induces the RS memory. In addition, this device has good thermal stability in the temperature range of 25 to 85 °C.

The human hair keratin with the configuration of FTO/keratin/Ag exhibits good RS performance, high transmittance, as well as physically transient properties (Figure 10m) [115]. The non-volatile memory performance exhibits a resistance window larger than 10^3^, switching ratio, and retention time of more than 10^4^ s. The keratin-based RRAM device can be dissolved within 30 min in deionized water. Cellulose can act as the functional layer material in the RS layer and it can be also used as a substrate to construct a flexible self-supporting memristor, which was studied by Xia et al. [119]. The cellulose membrane/Cr/Au/cellulose/Ag/Au device presents volatile and non-volatile RS behaviors by controlling the compliance current in the set process. Under a compliance current of 1.1 × 10^−7^ A, the device shows volatile threshold switching behavior with a set voltage in the range 0.2–0.6 V. The non-volatile bipolar switching behavior can be seen with the compliance current of 1.0 × 10^−4^ A, causing it to exhibit an ON–OFF ratio of 10^6^ and retention time of more than 1000 s. The device can work stably in the bending mode and in the temperature range from 20 to 100 °C. This study provides a facile strategy for constructing a natural polymer-based RS device.

Recently, Sun et al. studied the corn-starch-based biomaterial flexible RS device [116]. The corn starch was extracted directly from corn plants and mixed with PVDF to prepare a flexible ITO/corn-starch:PVDF/Ag sandwich structure. The device shows a capacitive effect at different values of *V*_max_. Notably, at the memory window of –12 V, the largest switching ratio is 3.5 × 10^2^, and almost symmetrical capacitive-coupled memristive *I–V* characteristics are achieved. By applying the appropriate pulse amplitude, width, and frequency, the device also presents synaptic behavior due to the presence of a large number of conducting ions in the active layer. The memristive device array may be used to map shape changes caused by subtle pressure changes through measuring the current changes, as schemed in Figure 10n. A word, “WATERLOO”, can be sensed by writing it with a stylus pen to record the current changes of the device cells. This work elucidates the role of corn starch in realizing eco-friendly green wearable electronics and its potential application in artificial intelligence.

## 6. Summary and Outlook

In conclusion, this review demonstrates a summary of recent progresses of polymer-based RS memory devices, including single-component polymers, polymer mixtures, 2D covalent polymers, and biomacromolecules. The advantages and disadvantages of these devices are listed in Table 1. The device structures, switching types, and mechanisms are thoroughly introduced. Based on these enlightening investigations, we have faith that the polymer-based RS materials and devices will open novel opportunities in the information storage and information processing fields to realize multifunctional memristors in future RS technology.

Generally, designing redox-active entities with donors and acceptors in polymer molecules is a popular strategy for achieving switching behavior. In order to obtain high –storage-capacity devices, a multilevel or ternary memory device is urgently needed. In addition to the traditional compliance current control in the device testing, designing a donor–acceptor–acceptor structure provides an innovative insight to realize ternary memory. Moreover, the unique properties of recently developed 2D covalent polymers with high chemical and thermal stabilities, large surface areas, and tunable electronic properties, renders it easy to obtain high ON–OFF ratios, low power consumption, and high stability memristors. Biomacromolecule-based RRAMs show interesting applications in mimicking the human brain, implantable devices, memory storage, and wearable electronics. They open a new window for the development of wearable and implantable memory devices.

Although tremendous advances have been witnessed in the investigation of switching polymer materials, there are still some fundamental issues that need to be conquered for further development of polymer switching. Firstly, to date, although the switching parameters (e.g., ON–OFF ratio, operational voltage, cycling endurance, and retention time) have been greatly improved by various methods, they are not very competitive with inorganic counterparts. The large cycle-to-cycle and device-to-device variations in the switching parameters result in low production yields of devices on crossbar arrays. This is far from valuable integration into large-scale integrated circuits. The fabrication technology of polymer memory devices is not mature enough to compete with the existing silicon-based memory devices for high reproducibility and low-cost production. Especially in 2D covalent polymers, achieving high-quality thin films with good reproducibility and crystallinity remains a challenge. Secondly, the stability of polymer memory devices is readily influenced by ambient moisture and temperature. For example, 2D covalent polymers and biomaterials are sensitive to moisture, light, and heat. There is still a long way for polymer materials to go with regards to commercial application, which requires more research efforts to optimize. Thirdly, some switching mechanisms have been proposed to account for the polymer materials. However, a definite mechanism with both theoretical and experimental evidence is not yet completely accepted. A deeper understanding of the different types of mechanisms is essential for molecular structure design and modification. For example, the charge transfer is a well-known switching mechanism in donor–acceptor structure. However, direct physical evidence is lacking to confirm the existence of a charge transfer state. Its long lifetime in a memory device is in conflict with the transient spectroscopy measurements performed on solar cells. Only with a clear understanding of the memory mechanism to guide the rational molecular structure–property relationship can the polymer memory device make rapid progress in the future.

Emerging RS memory devices based on polymer materials have advanced rapidly over the past years, through addressing the aforementioned scientific and technical issues. This research needs physicists’, chemists’, material scientists’, and electrical engineers’ infinite efforts in the field of polymer materials and devices in the upcoming era of artificial intelligence.

## Figures and Tables

**Table 1:** Summary of the advantages and disadvantages of different polymer-based RS memory devices.

	Advantages	Disadvantages
Single-component polymers	Easy molecule design. Avoids the probability of phase separation.	Switching performance is restricted. Complex synthesis route.
Polymer mixtures	Electronic properties, processability, and mechanical flexibility can be easily adjusted. Facile thin film preparation.	Phase separation.
2D covalent polymers	High thermal and chemical stabilities. Excellent scalability.	The quality of the thin film is limited. Slower write speeds.
Biomacromolecules	Self-decomposition, biodegradability. Environmentally friendly. Flexible.	A short life span. Complex purification of biofilms. Poor thermal stability.

## Data Availability

Not applicable.
